# Autoregressive Affective Language Forecasting: A Self-Supervised Task

**DOI:** 10.18653/v1/2020.coling-main.261

**Published:** 2020-12

**Authors:** Matthew Matero, H. Andrew Schwartz

**Affiliations:** Stony Brook University; Stony Brook University

## Abstract

Human natural language is mentioned at a specific point in time while human emotions change over time. While much work has established a strong link between language use and emotional states, few have attempted to model emotional language in time. Here, we introduce the task of *affective language forecasting* – predicting future change in language based on past changes of language, a task with real-world applications such as treating mental health or forecasting trends in consumer confidence. We establish some of the fundamental autoregressive characteristics of the task (necessary history size, static versus dynamic length, varying time-step resolutions) and then build on popular sequence models for *words* to instead model sequences of *language-based emotion in time*. Over a novel Twitter dataset of 1,900 users and weekly + daily scores for 6 emotions and 2 additional linguistic attributes, we find a novel dual-sequence GRU model with decayed hidden states achieves best results (*r* = .66). We make our anonymized dataset as well as task setup and evaluation code available for others to build on.

## 1 Introduction

With the growth of social media, natural language processing has increasingly turned toward problems in the social scientific domains. Language has been used to predict personality disorders, political ideology, and mental health (Preotiuc-Pietro et al., 2016a; Iyyer et al., 2014; Coppersmith et al., 2014). However, such predictions are typically made cross-documents or -users rather than cross-time (forecasting the future).

Predictions across time can enable another level of applications for NLP in the social sciences. For example, knowing how someone’s mood may change next week can be a valuable insight into preemptively preparing mental health assistance. We may be able to better predict a substance abuse relapse or suicide attempt, or provide individual insights into the precipitators of such events.^1^ Applications also exist in other domains, such as correlating mood and personal buying habits, or predicting the mood of performers (e.g. professional athletes) as insight into how well they will perform next.

One of the challenges for doing temporal predictions based on language is the availability of time-series data. Most user-level datasets only provide one measurement, typically of a demographic or psychological trait (something that doesn’t change frequently). On the other hand, document-level data (such as that of most sentiment analysis) may provide information across time, but they are typically divorced from the individual. Alternatively, instead of relying on annotations or self-reported data, one can simply predict the future language-based measurement (i.e. as output from another model).

Here, we propose a method of forecasting any *future* dimension of language, based on their *current and past* language. Formally, we define *affective language forecasting* as finding a function of past language (*f*) which estimates the output of a function of future language (*a*):

(1) $$f\left(X_{t-1}^{d},X_{t-2}^{d},\ldots ,X_{t-n}^{d}\right)+\epsilon =a\left(X_{t}^{d}\right)$$

where *X* represents a d-dimensional feature vector derived from language use at a particular time-step and *a* is conceptually a mapping of future language to the characteristic of interest. *ϵ* represents an error to be minimized. We show work towards this problem by forecasting multiple different dimensions of affective language. Affective language can be defined as any language that is related to mood or emotions, such as measuring affective valance(positive/negative mood) or something more specific such as the emotion of surprise. This work covers the forecasting of: Affective valence, Intensity, Anger, Disgust, Joy, Fear, Sadness, and Surprise.

Inclusion of the time dimension to an NLP problem can increase its modeling complexity significantly. We lay a modest foundation for handling these complexities, such as, the amount of history needed at the user level and what formatting of data needs to be done to facilitate modeling accurate predictions(i.e. time-step resolution). We also address the issue of gathering a large time-series dataset that tracks dynamic information at the user level.

**Our contributions include:**

- The definition of the task of *self-supervised affective forecasting* of lexicon frequency and the release of our novel dataset to promote community participation^2^
- Analysis of the characteristics of language that exist over the temporal dimension, notably related to the amount of history necessary to generate accurate forecasts of future linguistic trends
- Evaluation of modern deep learning techniques and their application or modification to the domain of time-series language forecasting
- Introduction of a new validation paradigm for time-series forecasting, making a distinction between an out-of-time forecast and users who are out-of-sample

## 2 Related Work

Time-series forecasting has been applied to many fields and applications. Traditionally, linear autoregressive(AR) models have been used in areas such as weather (Hillmer and Tiao, 1982) and stock forecasting (Pai and Lin, 2005). Similarly, AutoRegressive Integrated Moving Average(ARIMA) models are also popular choices in practice for imparting *stationarity*^3^ (Franses, 1991) to the time-series, specifically from the integration(I) step.

However, in recent years model architectures such as LSTM (Hochreiter and Schmidhuber, 1997) or GRU cells (Chung et al., 2014) have shown promise with their ability to model long-term dependencies. The recurrent nature of these models allow them to adapt to a time-series forecasting domain. Where input features are often lagged values of the predicted outcome, while still offering a more flexible architecture and non-linear modeling capabilities.

Such architectures have been used extensively in the medical domain, by leveraging electronic health records, to forecast a patient’s future physical health. For example, the work of (Choi et al., 2016) designs a stacked GRU architecture to forecast the diagnosis of a patient at their next doctor’s visit, while jointly learning to predict the duration until the next visit(an example of a poison process).

In the language domain the only similar work is that of (Halder et al., 2017). In which, their goal is to predict an online forum participants ”emotional progression” measured by their *negative emotion index*, a simple metric that defines the polarity of a user’s word count $\left(\frac{num(neg\_\text{words})-num(pos\_\text{words})}{num(\text{total}\_\text{words})}\right)$. We differentiate our work by focusing on forecasting established psychological definitions of affect and by using only linguistic features to forecast future linguistic trends.

## 3 Data and Time-Series

We derived our data set from a collection of publicly posted tweets previously used for demographic prediction (Volkova et al., 2013; Sap et al., 2014).

As one of the first studies of language forecasting of any kind, we sought to primarily focus on a well-represented time resolution – weekly aggregates of tweets. This helped us focus on the fundamentals of the methods rather than missing data issues.^4^ Only tweets without URLs (i.e. no retweets) were considered and users were filtered based on having 3+ original tweets per week, resulting in a dataset containing 850,000 tweets spanning 1,900 users. Our dataset contains users who posted between the years of 2009 and 2014 and had 20 contiguous weeks of data.

Additionally, we treat the problem as that of predicticting future *change* in affective language as opposed to modeling the *static* scores. By focusing on the change, the data is essentially detrended as if mean centered (i.e. increasing *stationarity* in our time-series) while the static scores can still be derived. To determine the change, a difference is taken between neighboring time steps for all features. After which, 19 data points remain in our sequence which is further split into 14 weeks for training and 4 weeks for testing per user.

### 3.1 Linguistic Dimensions

#### Affect and Intensity:

As our primary emotional dimension, we focus on affective valence. Valence, in conjunction with *intensity* or arousal, are often considered fundamental coarse-grained constructs for modeling emotional states (Russell, 1980; Posner et al., 2005). Valence can be thought of as the extent of positive or negative emotion. We use the pre-trained model valence and intensity of (Preoţiuc-Pietro et al., 2016b).

#### Six Emotions:

To model the language of finer-grained emotions beyond just positive/negative valence, we include the basic 6 emotions (Ekman, 1999). These consist of the following: Anger, Disgust, Joy, Fear, Sadness, and Surprise. We extract these emotions from text using a human curated lexicon, as defined by the NRC (Mohammad and Kiritchenko, 2015). The lexicon leverages the amount of words that display each emotion per message to measure each emotion.

#### Embeddings:

Language is also directly modeled as covariates to our psychological attributes in the form of word embeddings. 2 different sets of representations are learned from our data in an effort to evaluate which works better for temporal tasks. First, we learn 50-dimensional word2vec embeddings over a large corpus of social media data (15 million tweets). Second, we extract BERT representations (Devlin et al., 2019) for our tweets using bert-large and concatenate outputs from the last 4 layers. After extraction of BERT features, a non-negative matrix factorization (Févotte and Idier, 2011) is learned and applied using a subset of 50,000 tweets stratified across users. This matrix factorization brings the BERT features down to 50-dimensions to match word2vec. Embeddings are averaged across all words from a given week from a user. Models that leverage these additional context features are referred to as *multivariate*, as opposed to pure autoregressive(*univariate*) models.

### 3.2 Daily data

To consider the effect of temporal resolution and sparsity of data, a setup over days rather than weeks was also devised. This poses two interesting problems: (1) most users do not tweet in consecutive days leading to some missing data and (2) daily emotions are more prone to large fluctuations compared to longer stretches of time. The setup of the data is the same as the weekly counterpart, except that if a user does not have data for a given day it is marked empty for the models that can handle missing data (see GRU-D) or as no change, otherwise. These days are also excluded from testing data (so only days that had tweets are tested against). For daily resolution steps only a single linguistic dimension is explored: Affective valence.

### 3.3 Self-supervision

A key feature of affective language forecasting is its ability to be trained with self-supervision. Thus, we propose using the inherent structure of the language itself as the forecasting target. Much like how a language model will use the next observed word as its target we will use the next “observed“ attribute of language, as determined by a pre-trained model (Preoţiuc-Pietro et al., 2016b).

Importantly, the ratings used by Preotiuc-Pietro et al had high inter-rater agreement (*icc* = .77) and the models themselves were quite accurate in terms of producing continuous-valued scores(*r* = .65 and *r* = 0.80).

When not leveraging a pre-trained model, a hand curated lexicon is used to extract the underlying emotional target. As described for the 6 NRC emotions.

### 3.4 Train-Test Setup: Time and Users

In training a k-fold validation(k=5) is also performed, where for each fold the following sub-splits are generated: train users(weeks 1–14), test users 1 (weeks 15–19 of train users) referred to as *out-of-sample time*, test users 2 (weeks 15–19 of users held-out from training/validation) referred to as *out-of-sample users*, and validation users (weeks 15–19 of users held-out from training, different than test users 2).

In more detail, for train users only their first 14 weeks are used to train our models. The remaining weeks of these users form our main test set(in-sample user, out-of-sample time(**OOS Time**)). Validation users are out-of-sample users where only weeks 15–19 of their time-series is used to tune hyperparameters. Lastly, our second test set is comprised of weeks 15–19 from users excluded from both training and validation(out-sample user, out-of-sample time (**OOS User**)). This is because in practice, there could be applications where users will not be available for training but only for testing.

## 4 Methods

We seek to model the affective score of future language (*Y*_*t*_; e.g. the positive/negative valence of the language) as a function of past affective language (*Y*_*t*−1_*, Y*_*t*−2_, …) as well as other dimensions of past language (*X*_*t*−1_*, X*_*t*−2_, …; e.g. vector embeddings):

(2) $$Y_{t}=f\left(\left[Y_{t-1};X_{t-1}\right],\left[Y_{t-2};X_{t-2}\right],\ldots ,\left[Y_{t-k};X_{t-k}\right]\right)+\epsilon$$

where *t* represents the next time-span (e.g. next week, or tomorrow) and *k* represents a history length (order) of data to look backwards. All observations are over users.

This self-supervised objective, assumes affect scores at a given time point can be determined based only on the language at that time (i.e. *Y*_*t*_ = *a*(*X*_*t*_) where a is a model or mapping from language at time t to affect score). For example, a simple affect score may come from a lexicon of positive words which always produces the same affect score given the same set of word counts. On the more sophisticated side, the score may come from a complex predictive model based on the writings of the particular user at a particular time.

### 4.1 Dual Sequence, Decayed, GRU Network

Our primary approach is motivated by adapting powerful autoregressive sequence models for words and phrases (recurrent neural networks) to our task of forecasting affective language across weeks or days.

(3) $${\hat{Y}}_{t}=f\left(g\left(h_{t-1},Y_{t-1},X_{t-1}\right)\right)$$

where *Y* and *X* are the given affect scores and vector embeddings and *h*_*t*−1_ is the hidden state from the previous time step:

(4) $$h_{s}=g\left(h_{s-1},Y_{s-1},X_{s-1}\right)$$

*f* is the aggregation and activation function for the output layer and *g* is the composition and activation function for the recurrent layer.

Alternatively, one could model the progression as an aggregation over 2 separate, but related, sequences.

(5) $${\hat{Y}}_{t}=f\left(g_{1}\left(h_{t-1}^{\left(1\right)},Y_{t-1}\right),g_{2}\left(h_{t-1}^{\left(2\right)},X_{t-1}\right)\right)$$

(6) $$h_{s}^{\left(1\right)}=g_{1}\left(h_{s-1},Y_{s-1}\right)$$

(7) $$h_{s}^{\left(2\right)}=g_{2}\left(h_{s-1},X_{s-1}\right)$$

where each *g*_*n*_ is their own recurrent model learning from separate sequences of the affect score and other language dimensions. *f* now aggregates their learned representations and generates a prediction. A simple *f* would be to average the 2 separate predictions from each sequence.

#### GRU Setup:

We started with a standard GRU based RNN, due to their similar performance to LSTMs while requiring fewer computations, but change a few initial configurations to better reflect the characteristics of the autoregressive models rather than the typical sequential language tasks where GRU is mostly applied. A shared learnable initial hidden state parameter was added to the network, allowing the network to focus less on hidden state calculations at early time steps through the sequence. Additionally, all of the reset gate biases were initialized to −1, favoring retention of hidden states.

An attention layer is also included to allow the GRU to weight time-steps. The attention calculation is shown in equations 8, 9, and 10. Notice that in this context attention acts as a way of highlighting which time-steps may contribute more to mood fluctuations and the score itself is based on the hidden states of the other weeks. The first 2 equations handle the calculation of our attention weights for each time-step and the third is a weighted sum of the hidden states.

(8) $$\hat{H}=\text{tanh}\left(H_{1:t}\right)$$

(9) $$A_{t}=\text{Softmax}(W*\hat{H}+B)$$

(10) $$\text{attn}=\sum A_{t}*H_{t}$$

#### Decayed GRU:

GRU-D (Che et al., 2018) has two important differences over a traditional GRU in time-series tasks. First, it can account for missing data that is common across many time-series datasets. Secondly, it applies a (learned) decay parameter across hidden-states to better emulate the passing of time. An overview of these decays are below: 11 shows the formula for the learnable decay parameters where *δ*_*t*_ is a time interval vector which encodes the time gap between observations, 12 shows the parameter applied to the input layer where $m_{t}^{d}$ is a binary masking vector for variable *d* at time *t*, ${\tilde{x}}^{d}$ is the mean, and $x_{t^{\prime }}^{d}$ is the last observed value of *x*^*d*^ and 13 is the decay applied to hidden states.

(11) $$\gamma_{t}=\text{exp}\left(-max\left(0,W_{\gamma }\delta_{t}+b_{\gamma }\right)\right)$$

(12) $${\hat{x}}_{t}^{d}=m_{t}^{d}x_{t}^{d}+\left(1-m_{t}^{d}\right)(\gamma_{x_{t}}^{d}x_{t^{\prime }}^{d}+\left(1-\gamma_{x_{t}}^{d}{\tilde{x}}^{d}\right)$$

(13) $${\hat{h}}_{t-1}=\gamma_{h_{t}}⊙h_{t-1}$$

#### Dual-Sequence:

**Figure 1:**
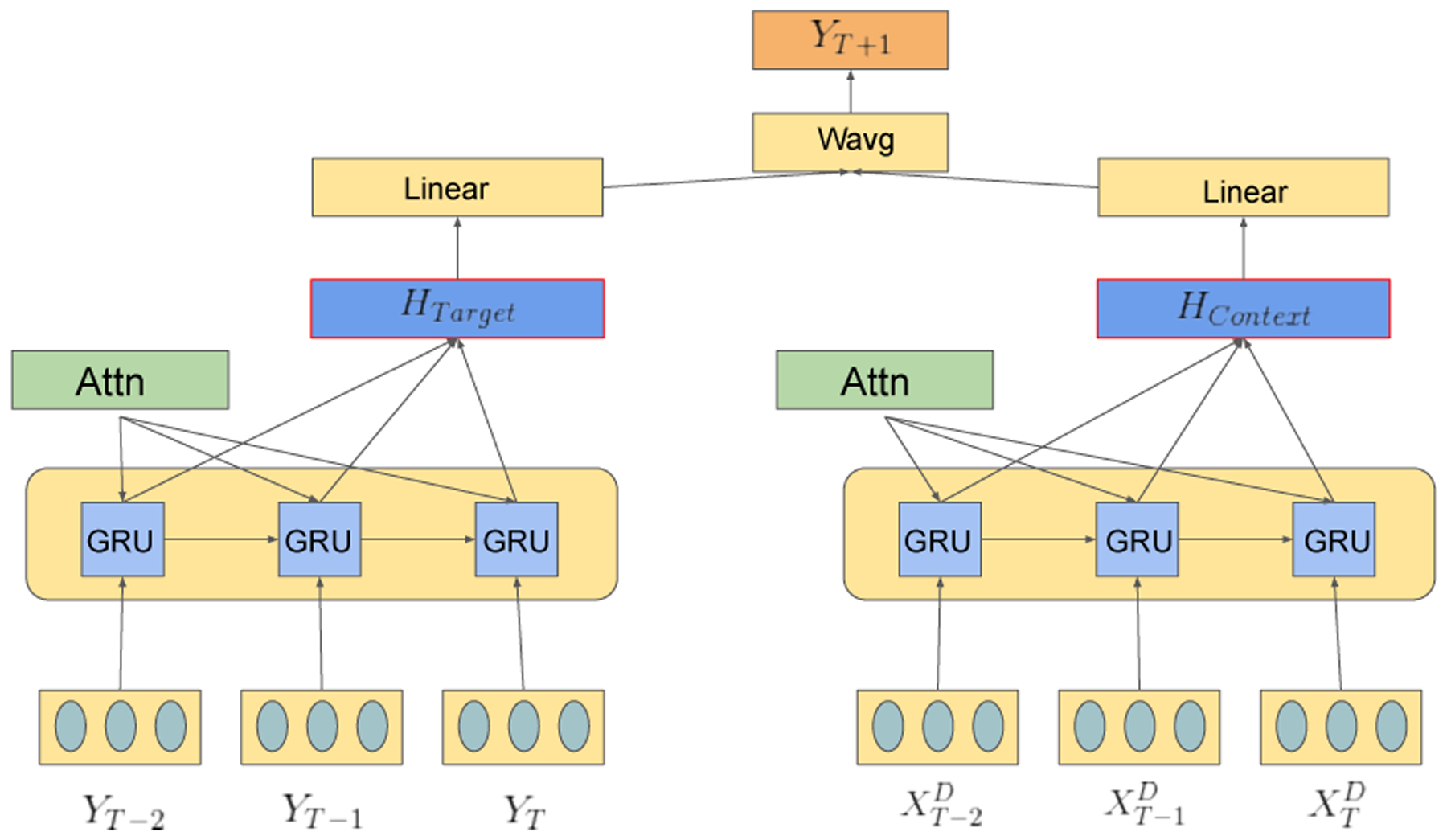
Architecture of parallel dual GRU system. Each GRU has its own attention weights, the red border denotes tanh activation across hidden output. Wavg is a weighted average where weights are learned through training with an initial configuration of .6/.4 target/context respectively

In the models thus far, the GRU does not explicitly handle the input dimension representing previous outcomes (*Y*_*t*−1_) any different than any dimension of the input embedding. Thus, we also developed another architecture that utilizes 2 parallel GRU layers. In this configuration, one GRU layer will perform a pure(univariate) autoregression where its only input will be the affect score(affective valence, intensity, etc) and the other GRU will take in any context variables(embeddings). This approach enables separate attention layers, possibly highlighting different time-steps depending on the type of data, while still training simultaneously. Structure of our proposed model is shown in figure 1, 2 GRUs with their own attention layers and their final prediction being a (learned) weighted average of their outputs.

#### Dynamic Model Order:

**Figure 2:**
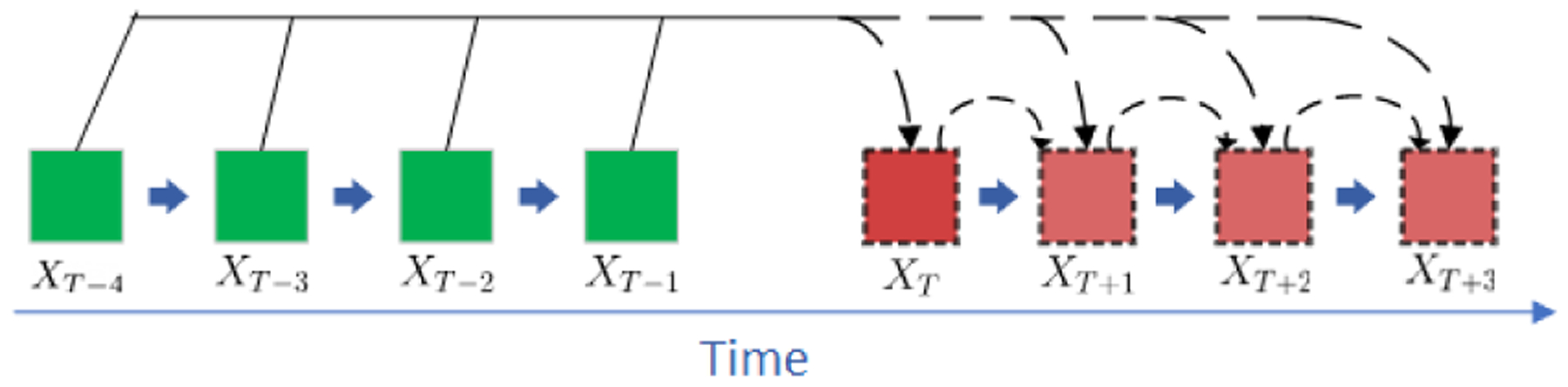
Visualization of an autoregressive prediction with an order *p*=4. The first prediction is made using only the green(previously observed) data. For each proceeding prediction the lag moves forward 1 time-step, but still maintains 4 previous observations as input.

Normally when training an autoregressive model a restriction is set to apply a fixed number of lag observations, commonly referred to as the *order* of regression denoted as *p*. The order *p* is the amount of language history added to the model to make a prediction. Structure of an autoregressive model can be seen in figure 2.

Due to the dynamic unrolling nature of RNNs, there is no restriction to training with a set fixed *p*. Here, we introduce dynamic order training: generating training examples of various length sequences for each user with a selected minimum order and then add 1 more observation for each new history length. Resulting in a training set where the first example is taken from each fixed order model(use weeks 1–3 to predict 4, 1–4 to predict 5, until reaching the maximum 1–14 to predict 15). Both fixed and dynamic *p* models are explored in the context of GRUs.

For models that can directly handle missing data(GRU-D) we mark dynamic *p* sequences shorter than the maximum length(14) as missing. When using a fixed *p* we run GRU-D in a slightly different configuration, namely **GRU-DS**, which only accounts for the internal state decay over time and doesn’t directly model the missing data on the input layer.

### 4.2 Alternative Approaches

#### Deep Averaging Network:

One way to model this problem is to ignore the sequential information entirely but model the non-linearity’s between the average change in dimensions over a time-span. However, this approach is naive and is not encoding the most important aspect: time. A deep averaging network(DAN) (Iyyer et al., 2015) with 2 dense layers and sigmoid activation between them is used. During univariate training there are only 2 hidden units at each layer, while there are 35 when doing multivariate.

#### Support Vector & Gradient Boosting Regression:

These 2 models are popular approaches for many machine learning tasks (Yoshimi and Kotani, 2019; Liu et al., 2019). The SVR chosen uses a linear kernel and both models are implementations from the sklearn python package (Pedregosa et al., 2011).

#### Autoregressive Ridge (AR-Ridge):

A L2-regularized(ridge) linear regression is also considered. In the field of time-series analysis a traditional linear regression is often preferred for these types of tasks (Lydia et al., 2016; Balakrishna et al., 2018).

#### Baselines: Simple Moving Average and Constants:

As a new task we propose a collection of heuristic baseline models. For example, to predict the future one may reuse values from the past(i.e. someone’s change in mood may be identical to their change last week).

We employ standard “zero rule” baselines, with some modifications to time-series. These are intended to capture easy to implement or naive approaches for prediction (Andrzejak et al., 2008; Miglionico, 2019). The following 2 baselines are considered: (1) predict the last time step’s change (**last(1)**) and (2) average of all available changes over a 14 time-step history (**mean(14)**).

An additional third baseline could be used where one would predict 0 change (**no change**), However, for our evaluation we stick with the previously mentioned 2 baselines due to using *Pearson correlation (r)* as our main evaluation metric. Where predicting 0 change would have no correlation with the actual time-series values. Correlation is chosen as it is resistant to the scale of the target variable. Thus allowing direct comparison of difficulty in forecasting across all of our affective dimensions

#### Transformer Networks:

A popular approach to many tasks in Natural Language Processing is to *fine-tune* a pre-trained language model (i.e. BERT) rather than train a smaller model from scratch. However, these language models are pre-trained over individual messages and would require alternative architectures for aggregating over multiple messages and capturing time. Since we are introducing the problem we focus on a more fundamental setup and suggest such architectures would be an interesting direction for future work. For example, one could explore various architectures for hierarchical models on top of standard pre-trained transformer language models.

##### Ethics Statement:

This research has been approved (deemed exempt status) by an academic institutional review board.

## 5 Evaluation

The first set of results showcases the chosen zero rule baselines compared to all deep learning models using a fixed order of 14 (the max). We show the results for the test set consisting of **OOS Time** users for target variable affective valence. The zero rule baselines perform quite well, showing that even out-predicting the past can be challenging. Note that the deep averaging network is operating on the same information as the **mean(14)** baseline, so we expect it to perform similarly.

Additionally in practice there are many users who would be forecasted but would possibly not be part of the training data of any particular model. To emulate this we examine results for a set of held-out users for which our model has neither trained nor been tuned on. We compare the results across select models from the previous test set and this **OOS User** test set in table 2. There seems to be no loss in predictive power when evaluating against this held out set; showing the robustness of the proposed models when fitting to a real application scenario.

**Figure 3:**
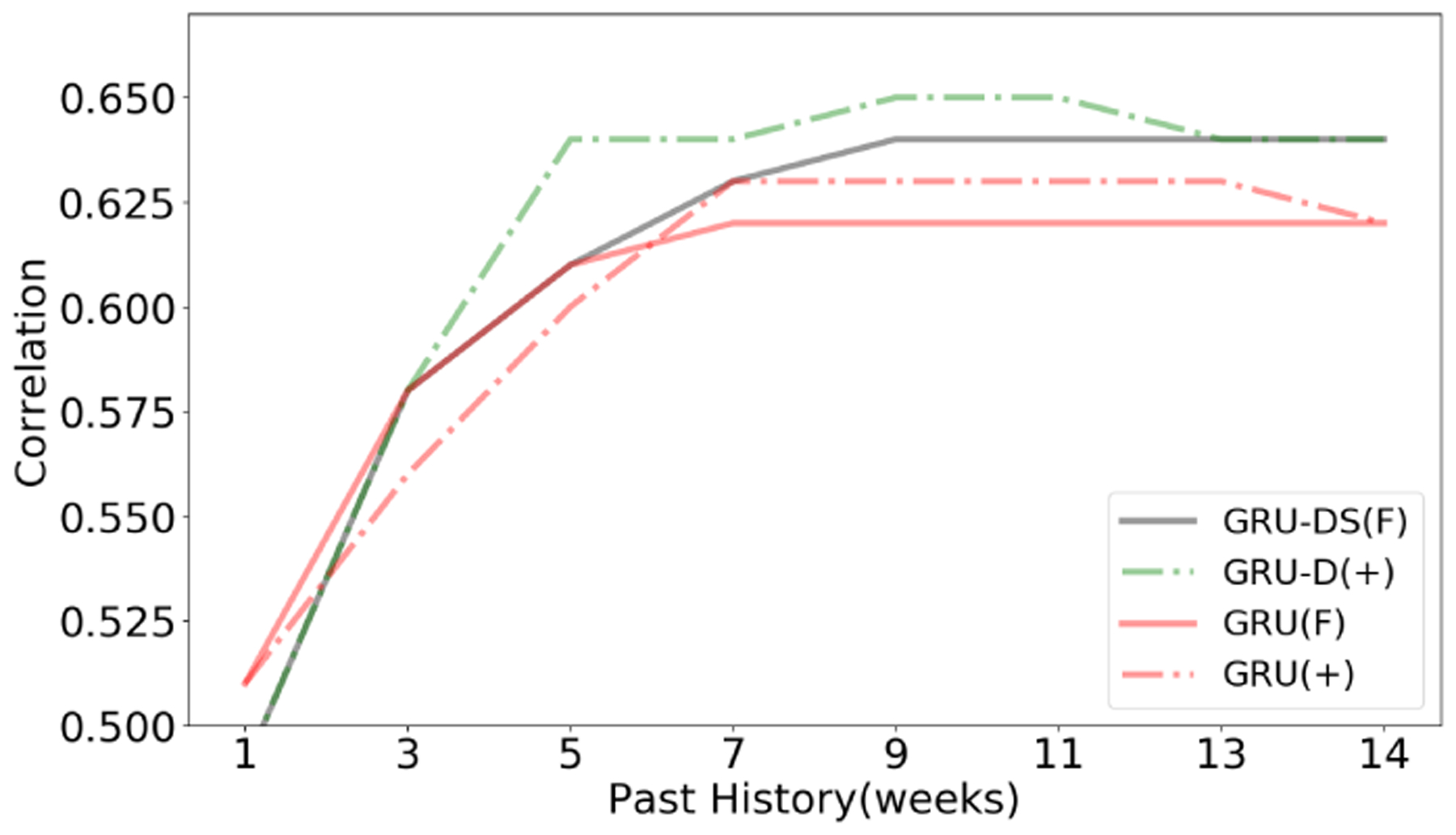
Comparison of GRUs for fixed and variable order pure autoregressive approaches as more history is added to the training examples for target variable affective valence. GRU-D always leverages 14 weeks of data (Where anything older than the set order is marked as missing and leverages the mean instead). + denotes dynamic order and F denotes fixed order.

In figure 3, we show the effect of order *p* on our autoregressive models with respect to correlation in a pure autoregressive setup. As mentioned, a benefit of using recurrent neural models for time-series modeling is the ability to implement dynamic unrolling on variable length sequences. To explore the effectiveness of our dynamic order technique for time-series, we directly compare the GRU models using variable *p* against training with a fixed *p*.

We find that applying the dynamic order to a model such as GRU-D, where data can be marked as missing, gives clear benefits when compared against smaller fixed orders. However, as the order size increases this loses its effectiveness. With respect to the amount of *p* required to create a robust model, we typically find that most models perform best when utilizing 9 weeks of *p* before saturating.

We believe this is due to the shrinking of the training set. For each extra time-step of history, there is a loss of 1 training example per user. In other words, each increase of 1 time-step of *p* reduces training examples by approximately 1,900.

In table 3, we compare results across models when word embedding features are also included. From our results we can see that utilizing the dual-GRU setup where one GRU handles a univariate autogression and the other handles context variables is generally more favorable than utilizing only a single(joint) GRU. However, generally there seems to be difficulty in extracting useful temporal information from language representations in most cases.

We also highlight the differences in performance between using word2vec and BERT representations in table 4. Typically, word2vec performs better than BERT for this task. We believe this is due to 2 reasons: (1) the objective of word2vec is more closely related to the way the pre-trained models were trained(BOW) and how lexicons judge emotion (word occurrence, not context) and (2) BERT may be too high of a level of semantics when trying to forecast the underlying temporal characteristics of language.

Affective valence prediction is also examined using off-the-shelf models such as SVR, GBR, and (ridge) autoregression. Here, we show the best performing variants of each(their optimal *p*) and compare to our best GRU model(dual GRU-DS *p*=13), shown in table 5.

**Figure 4:**
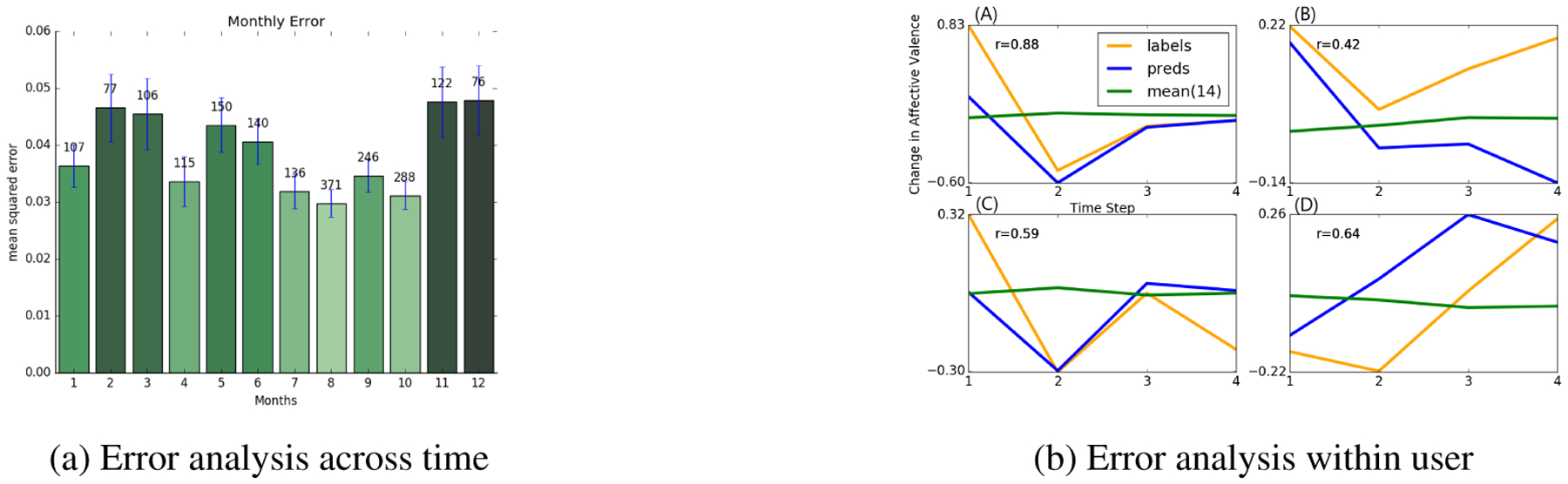
(A) Average squared error per month across our OOS Time set; shown with standard error bars and the amount of users in that month. Higher error is related to having fewer users that fell into that test month rather than influence from any seasonal event(i.e. winter holidays or summer vacations). (B) Dual GRU-DS performance when evaluating against a user’s change in affective valence across weeks, compared with baseline mean(14) and the ground truth labels. We find that our predictions are typically accurate. However, even when they fail the general trend is still present

A robustness check is performed to analyze sensitivity to seasonal trends. Figure 4a shows the squared error averaged across all years of data separated by month for affective valence. Notably, there is not a large increase in error during the winter months where one may expect emotions to be abnormal due to the many holidays that occur, but rather error seems more dependent on number of users present in that testing month, suggesting that this task scales well with respect to dataset size at the user level.

Robustness is also checked within user, where we visualize our model’s forecasted weekly changes for a person’s affective valence, shown in figure 4b. Our model achieves great success at not only producing accurate predictions but also capturing general trends in mood. However, there is evidence of the model struggling when a user continually has large changes in mood week after week.

Results for affective attributes listed in section 3.1 are shown in table 6. Similar trends to affective valence are seen across these attributes, implying there is an underlying structure of affective language in the temporal domain.

Finally, performance on the daily resolution dataset for affective valence is presented. A brief selection of results is shown in table 7. In this setup, we only utilize a pure autoregressive approach rather than multivariate, due to the abundance of missing data. GRU-D performs the best on this task, which we believe is due to the nature of GRU-D and its ability to model missing data directly.

## 6 Conclusion

We presented a new subtask in NLP, *self-supervised affective forecasting*. The models proposed in this work cover a wide range from traditional machine learning to deep learning techniques. While traditional models perform well, GRUs with decayed hidden states provided more accurate results and offer a more flexible architecture. We find this most helpful when experimenting with extra context features, such as word embeddings, splitting the inputs into 2 separate forecasts for a dual sequence GRU offers the best predictive power.

We also show that our modeling choices are robust to both seasonal variation and multiple evaluation paradigms: **OOS Time** and **OOS User**. Lastly, we found a strong relationship between the order of our models and the accuracy of future predictions with a performance leveling out roughly at a history of 9 weeks for pure autoregressive and 13 weeks for dual GRU-DS.

Affective forecasting can be leveraged for many domains, such as social sciences (mental health), business (consumer buying habits, targeted ads), and athletics (game performance). We encourage others to pick up this challenging task by releasing our novel dataset and task setup code.

## Figures and Tables

**Table 1:** Performance of proposed GRU models: GRU with attention, and GRU with memory decay (GRU-D, GRU-DS) compared with baselines and DAN. All models use the max, order of 14, fixed history (*p*) and run with the pure auto-regressive (univariate) input. Bold results are found significant with *p <* .05 using a paired t-test.

Model (order)	*r*
*Baselines*	
Last (1)	.47
Mean (14)	.49
DAN (14)	.49
GRU (14)	.62
GRU-DS (14)	**.64**
GRU-D (14)	**.64**

**Table 2:** Comparison of our various GRU model performance when using in-sample users, out-sample time and out-sample users, out-sample time. Uses pure auto-regressive setting (only history of previous outcomes).

Model (order)	OOS Time(*r*)	OOS Users(*r*)
GRU (11+)	.63	.63
GRU-D (11+)	.63	.64
GRU-DS (9)	.64	.64

**Table 3:** Pure auto-regressive versus contextual auto-regressive / Joint versus Dual: Comparison of univariate (pure autoregressive) versus multivariate (contextual – includes features beyond the target variable for the past) across the best models. Bold results show significance with *p <* .05.

Model (order)	Uni(*r*)	W2V(*r*)
*Joint Sequence*		
GRU (11+)	.63	.63
GRU-DS (13)	.64	.64
GRU-D (9+)	.65	.65
*Dual Sequence*		
GRU (11+)	-	.63
GRU-DS (13)	-	**.66**
GRU-D (9+)	-	**.66**

**Table 4:** Comparison between the different types of language representations, word2vec and bert across their best GRU configurations. We believe BERT does not directly capture what our self-supervision technique high-lights for emotions. Bold results show significance with *p <* .05.

Model (order)	W2V(*r*)	BERT(*r*)
*Dual Sequence*		
GRU (11+)	.63	**.62**
GRU-DS (13)	**.66**	.61
GRU-D (9+)	**.66**	.61

**Table 5:** Performance among traditional ML models given their optimal *p* and our best performing GRU model. The traditional models perform quite well offering simplistic use, however the improvement with deep learning implies there is more room to grow given larger data and model flexibility. Bold results show significance with *p <* .05

Model (order)	*r*
SVR (13)	.64
GBR (5)	.58
AR-Ridge (9)	.64
GRU-DS (13)	**.66**

**Table 6:** Results for the best model (dual GRU-DS) for all other linguistic emotion dimensions for **OOS Time** testing. In this configuration we used multivariate input(word2vec) and an order of 13 weeks.

Language Variable	*r*
Intensity(Arousal)	.63
Anger	.64
Disgust	.63
Fear	.65
Joy	.63
Sadness	.66
Surprise	.64

**Table 7:** Pure autoregressive **OOS Time** results across models at **daily resolution**. All models use a observation order of 3, which was found ideal, except the zero rule baseline which is 1 by definition. Bold results show significance with *p* < .05.

Model (order)	*r*
Last (1)	.22
SVR (3)	.36
AR-Ridge (3)	.37
GRU (3+)	.34
GRU-DS (3)	.37
GRU-D (3+)	**.38**

## Footnotes

1 Deaths from suicide are the second leading cause of death for young adults and overdoses are on the rise, not far behind (Curtin et al., 2016; Rudd et al., 2016)

2 Data available at https://github.com/MatthewMatero/AffectiveLanguageForecasting

3 Ensuring that our time-series maintains the same, approximate, statistical properties over time. Such as mean and variance

4 We also explored the, more sparse, daily level as a final experiment.
